# Systemic Analysis of Gene Expression Profiles Identifies ErbB3 as a Potential Drug Target in Pediatric Alveolar Rhabdomyosarcoma

**DOI:** 10.1371/journal.pone.0050819

**Published:** 2012-12-05

**Authors:** Janne Nordberg, John Patrick Mpindi, Kristiina Iljin, Arto Tapio Pulliainen, Markku Kallajoki, Olli Kallioniemi, Klaus Elenius, Varpu Elenius

**Affiliations:** 1 Department of Medical Biochemistry and Genetics, University of Turku, Turku, Finland; 2 VTT Technical Research Centre of Finland, Turku, Finland; 3 Department of Pathology, Turku University Hospital, Turku, Finland; 4 Institute for Molecular Medicine (FIMM), University of Helsinki, Helsinki, Finland; 5 Medicity Research Laboratories, University of Turku, Turku, Finland; 6 Department of Oncology, Turku University Hospital, Turku, Finland; 7 Department of Pediatrics, Turku University Hospital, Turku, Finland; University Hospital of Modena and Reggio Emilia, Italy

## Abstract

Pediatric sarcomas, including rhabdomyosarcomas, Ewing’s sarcoma, and osteosarcoma, are aggressive tumors with poor survival rates. To overcome problems associated with nonselectivity of the current therapeutic approaches, targeted therapeutics have been developed. Currently, an increasing number of such drugs are used for treating malignancies of adult patients but little is known about their effects in pediatric patients. We analyzed expression of 24 clinically approved target genes in a wide variety of pediatric normal and malignant tissues using a novel high-throughput systems biology approach. Analysis of the Genesapiens database of human transcriptomes demonstrated statistically significant up-regulation of *VEGFC* and *EPHA2* in Ewing’s sarcoma, and *ERBB3* in alveolar rhabdomyosarcomas. *In silico* data for *ERBB3* was validated by demonstrating ErbB3 protein expression in pediatric rhabdomyosarcoma *in vitro* and *in vivo*. *ERBB3* overexpression promoted whereas *ERBB3*-targeted siRNA suppressed rhabdomyosarcoma cell gowth, indicating a functional role for ErbB3 signaling in rhabdomyosarcoma. These data suggest that drugs targeting ErbB3, EphA2 or VEGF-C could be further tested as therapeutic targets for pediatric sarcomas.

## Introduction

According to the European Medicines Agency (EMEA) 50–90% of drugs have never been tested for use in children [1], [2]. Still drugs are frequently used for pediatric patients off-label, risking activity and raising concerns about adverse effects [3]. Several new cancer drugs targeting specific signaling molecules have recently been approved for clinical use [4], [5]. Little is known about their activities in pediatric patients, or about the presence of their target proteins in pediatric cancer tissues.

Pediatric sarcomas, including rhabdomyosarcomas, Ewing’s sarcoma, and osteosarcomas, are highly malignant tumors in children, adolescents and young adults, and account for approximately 6% of all childhood malignancies [6]. Sarcomas have a low survival rate despite multimodal treatment, including surgery and/or radiotherapy in combination with multidrug adjuvant chemotherapy. For localized disease, the overall 5-year survival rates approach 60–70% [7], but metastatic sarcomas have a significantly less favorable prognosis with 5-year overall survival ranging between 10% and 30% [8], [9]. The quality of life of long-term survivors may also be compromised [10]. Thus, innovative new therapies are needed.

Systems biological and computational approaches can benefit cancer research and drug development. Systems level high-throughput analyses of cancer samples generate an extremely wide range of data about somatic and germ-line mutations, as well as about cancer-specific transcript and protein expression profiles. Electronic databases can be used to explore gigabytes of data from cancer genomes, transcriptomes and proteomes (genes, messenger RNAs, proteins, and their modifications) that are generated in independent projects worldwide. Converting the knowledge obtained using these omics techniques into clinical applications may provide novel and more specific options for cancer therapy [11].

In this study, the Genesapiens *in silico* transcriptomics database (www.genesapiens.com) [12] including calibrated data from 9783 publicly available Affymetrix analyses was used to identify transcripts overrepresented in human pediatric sarcomas. Differential expression analysis of 24 target genes for clinically available therapeutic antibodies, tyrosine kinase inhibitors, and proteasomal inhibitors demonstrated specific up-regulation of transcripts encoding ErbB3 in pediatric rhabdomyosarcomas, and transcripts encoding VEGF-C and EphA2 in Ewing’s sarcoma. The data were validated by demonstrating ErbB3 protein expression in clinical rhabdomyosarcoma and suppression of rhabdomyosarcoma cell growth by RNA interference-mediated down-regulation of ErbB3. Thus, currently available targeted cancer drugs, such as inhibitors of the ErbB or VEGF receptors, or dasatanib with affinity for EphA2 [13], could be further evaluated as novel therapeutics for pediatric bone tumors. These data provide both candidates for novel drug targets as well as suggest novel indications for existing cancer drugs for the therapy of pediatric sarcomas.

**Figure 1:**
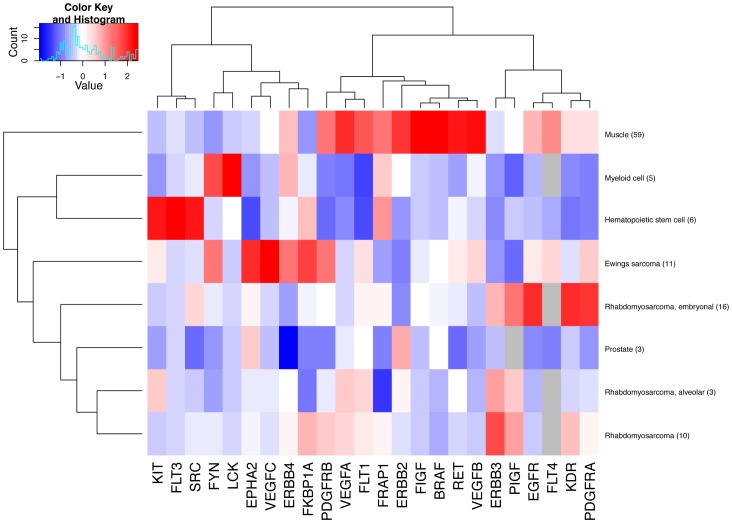
Hierarchial clustering of validated therapeutic target genes in rhabdomyosarcomas and Ewing’s sarcomas. The red boxes illustrate mRNA expression levels exceeding the mean expression per gene in the analyzed tissues, whereas the blue boxes illustrate mRNA expression levels lower than the mean expression per gene. The number of samples analyzed per tissue type is given in parentheses.

## Methods

### Affymetrics Data Collection

The Genesapiens *in silico* database of human transcriptomes was constructed by collecting data from 9783 publicly available Affymetrics microarray experiments in the form of CEL files as the source material (www.genesapiens.com). The data including arrays of normal and pathological human *in vivo* tissue samples were preprocessed and normalized, as previously described [12]. Altogether, the samples covered 15 pediatric cancer tissues (n = 1015) and 11 different pediatric normal tissues (n = 154). The normal samples represented hematological (n = 80), connective (n = 59), urogenital (n = 9), nervous (n = 4), and endocrine (n = 2) tissues. Samples from patients of 16 years or younger were defined as pediatric samples.

**Figure 2:**
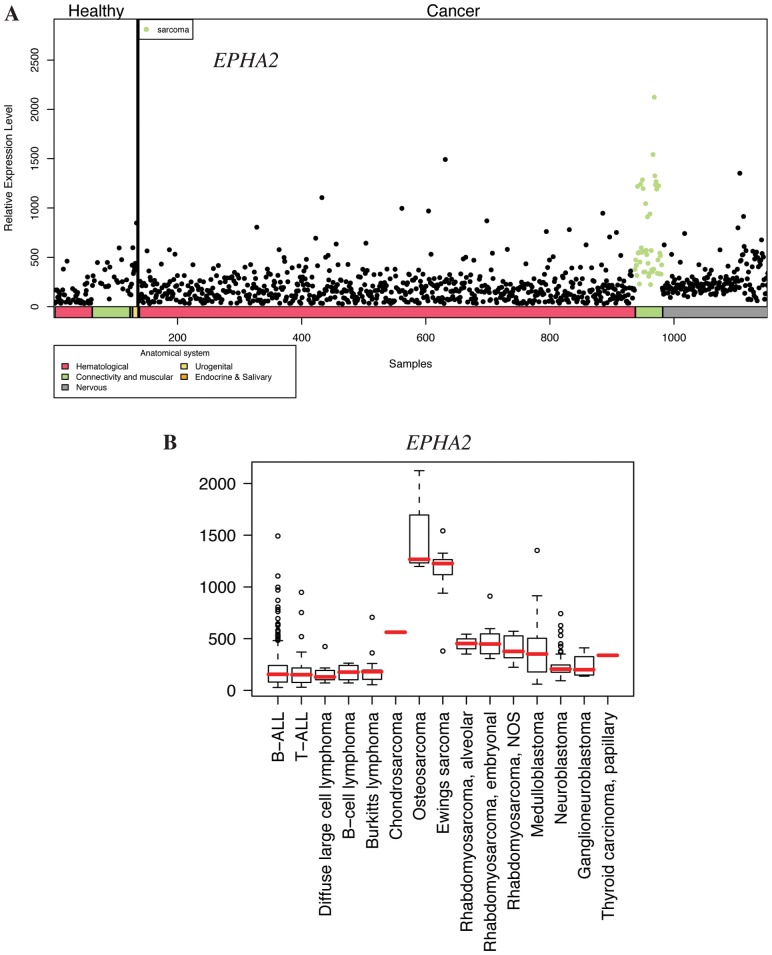
Expression profile of *EPHA2* in pediatric healthy and cancer tissues. A ) Body-wide expression profile of the *EPHA2* gene across the database. Each dot represents the expression of *EPHA2* in one sample. Anatomical origins of each sample are marked in color bars below the gene plot. The *EPHA2* gene is highly expressed in malignant connective and muscular tissue samples (green dots). **B**) Box plot analysis of the *EPHA2* gene expression levels across a variety of pediatric cancer samples. *EPHA2* is particularly highly expressed in osteosarcoma and Ewing’s sarcoma. NOS, not otherwise specified.

### Gene Expression Heatmaps for Clinically Relevant 24 Human Cancer Genes

Body-wide expression maps of 24 known cancer gene targets were generated with hierarchical clustering (Euclidean distance) of mean expression profile for genes across 4 human pediatric tumor subtypes (n = 40) of muscle or mesenchyme origin and 4 pediatric normal tissues (n = 73). The 4 control tissues were selected from the available pediatric data as they represented available primary tissues from which Ewing’s- or rhabdomyosarcomas have been clinically diagnosed or speculated to origin from [9], [14], [15]. Values for each gene across tumor and normal samples were mean centred at 0 with a standard deviation of 1. Tumor and tissue specific genes acquire a high score in the overall heatmap and can be identified in spots with a strong red color. Red color indicates increased level of expression. Blue color indicates decreased level of expression.

**Figure 3:**
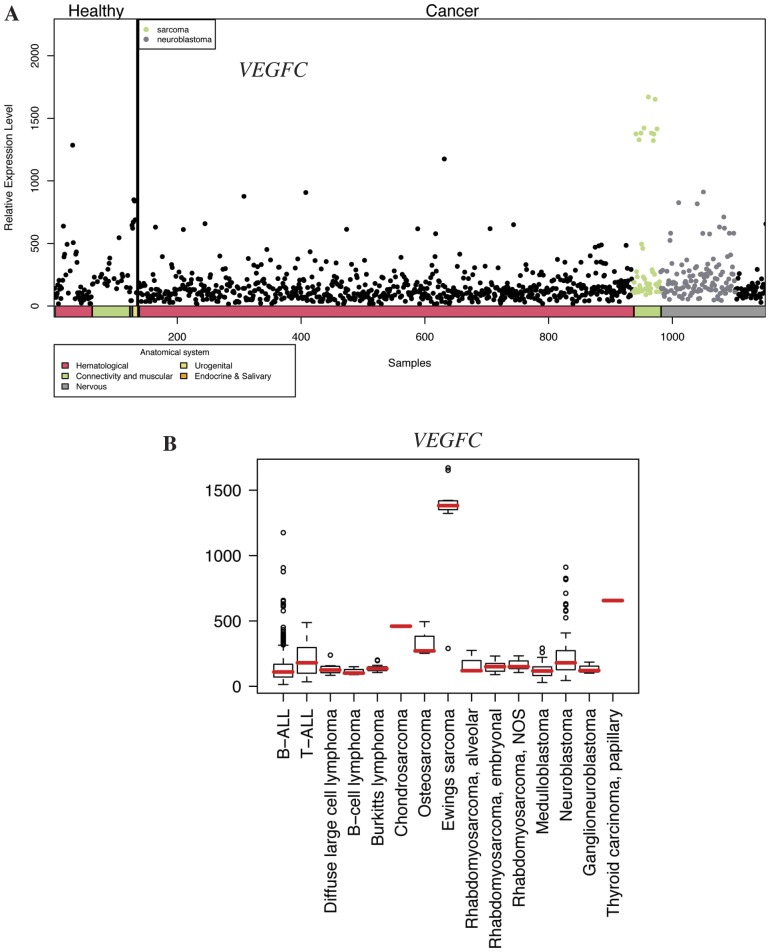
Expression profile of *VEGFC* in pediatric healthy and cancer tissues. **A**) Body-wide expression profile of the *VEGFC* gene across the database. Each dot represents the expression of *VEGFC* in one sample. Anatomical origins of each sample are marked in color bars below the gene plot. The *VEGFC* gene is highly expressed in samples originating from malignant connective or muscular tissue (green dots). The ten green dots forming a separate group with high *VEGFC* expression all represent samples from Ewing’s sarcoma. **B**) Box plot analysis of the *VEGFC* gene expression levels across a variety of pediatric cancer samples. *VEGFC* is particularly highly expressed in Ewing’s sarcoma. NOS, not otherwise specified.

### Body-wide Expression Profiles

The expression profile of a single gene across all pediatric tissues was visualized with custom designed body-wide expression plots. Body-wide expression profiles show the expression of a single gene at the level of individual pediatric samples, while its layout allows easy analysis of the biological or medical significance of the profile. The y-axis defines the expression level of the gene and the x-axis defines all samples arranged into fixed order by the type of sample (healthy, cancer) and subsequently by different tissue types (hematological, connective and muscular, nervous, urogenital and endocrine). Thus, each dot describes the expression levels of a particular gene in one sample. The anatomical origin of each sample is shown in the color bar at the bottom of the image. Tissues expressing the gene at more than one standard deviation higher than the baseline level for that gene across all samples are colored and displayed at the top.

**Figure 4:**
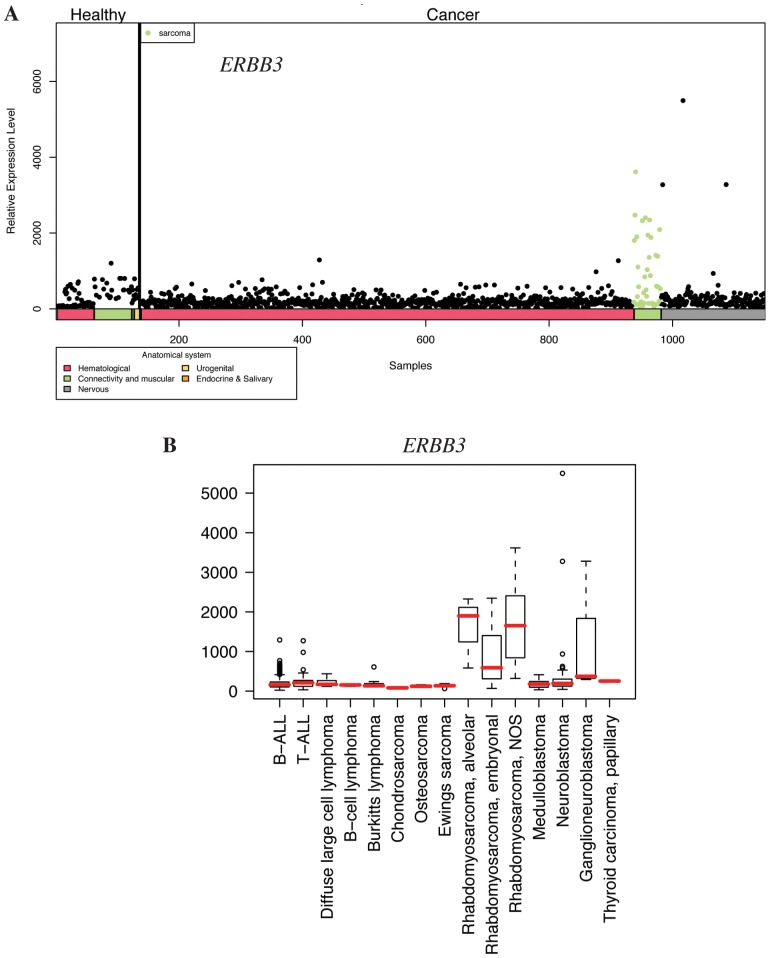
Expression profile of *ERBB3* in pediatric healthy and cancer tissues. **A**) Body-wide expression profile of the *ERBB3* gene across the database. Each dot represents the expression of *ERBB3* in one sample. Anatomical origins of each sample are marked in color bars below the gene plot. The *ERBB3* gene is highly expressed in malignant connective and muscular tissue samples (green dots). **B**) Box plot analysis of the *ERBB3* gene expression levels across a variety of pediatric cancer samples. *ERBB3* is particularly highly expressed in alveolar rhabdomyosarcomas. NOS, not otherwise specified.

### Boxplots

In the boxplots, the expression profiles of a single gene were displayed grouped into pediatric malignant samples (red boxes). All tumor samples for one tumor type were compared to all other pediatric samples (normal and cancer samples). The boxplots show the dispersion and skewness in the data. The data were split into five parts [lower quartile (Q1), median (Q2), upper quartile (Q3), and largest observation that is considered a non-outlier in a statistical sense] represented by the horizontal bars. The samples displayed above the last bar are considered outliers, representing data observations which lie more than 1.5*inter-quartile range higher than the third quartile.

**Figure 5:**
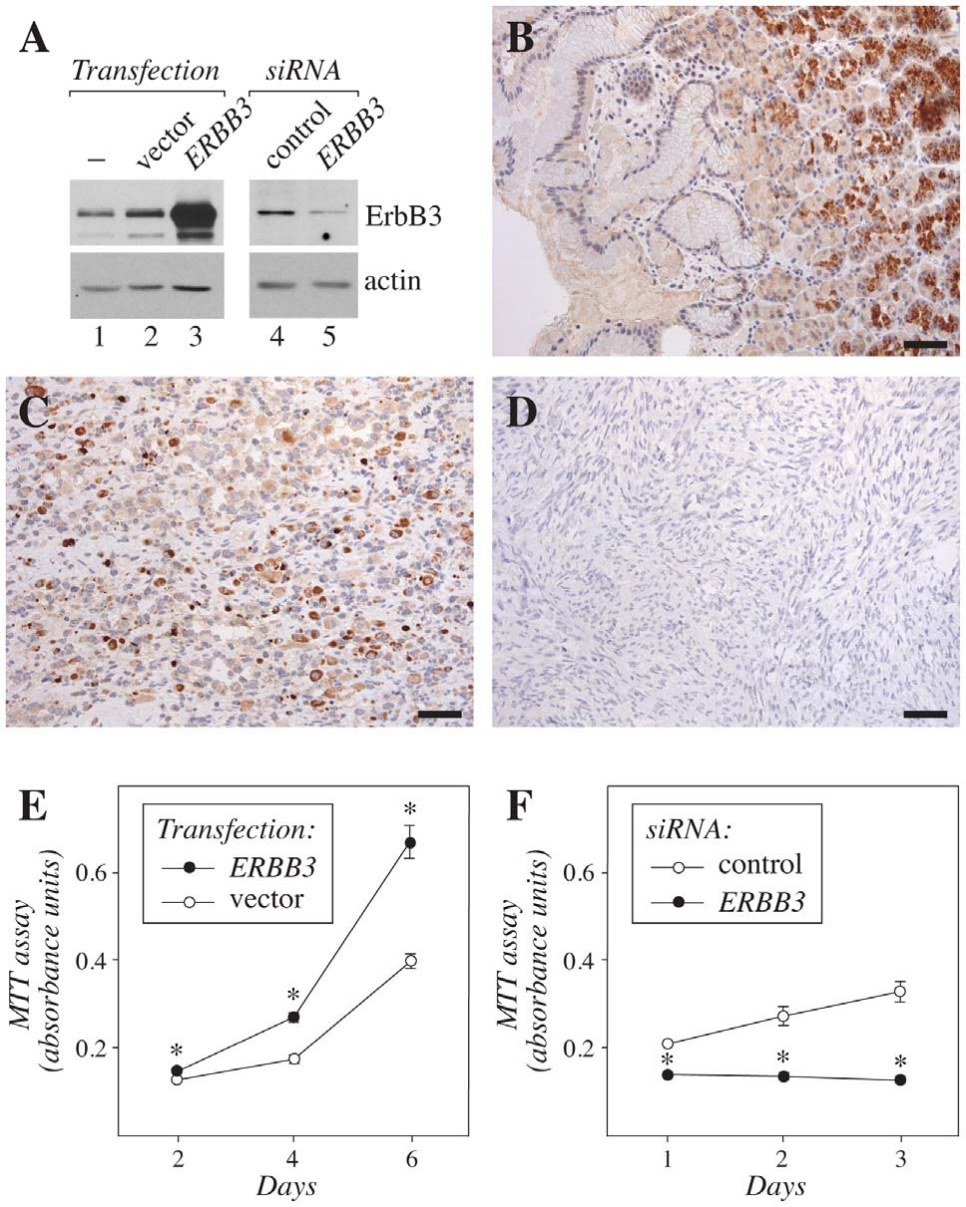
Expression and function of ErbB3 in rhabdomyosarcoma. (**A**) Western analysis of ErbB3 expression in RD cells transfected or not with a plasmid encoding ErbB3 or an empty vector control (lanes 1–3), or with *ERBB3*-targeting or control siRNA (lanes 4–5). Membranes were reblotted with anti-actin to control loading. (**B–D**) Immunohistochemical analysis of ErbB3 expression in normal adult human stomach (**B**) and in two samples representing pediatric alveolar rhabdomyosarcoma (**C**,**D**). (**E**,**F**) MTT proliferation analysis of the effect of *ERBB3* overexpression (**E**) or siRNA-mediated *ERBB3* down-regulation on the growth of RD cell transfectants. Expression of ErbB3 in the transfectants is shown in (**A**). *, *P*<0.001 when compared to control. Scale bar in (**B–D**), 100 µm.

### Plasmids, siRNAs, and Transfection

RD rhabdomyosarcoma cells (ATCC CLL-136) were transfected with pcDNA3.1*ErbB3* (insert includes nucleotides 277–4305 from GenBank accession NM_001982) or an empty pcDNA3.1neo(–) vector (Invitrogen) using FuGENE 6 (Roche). To down-regulate endogenous ErbB3 expression, RD cells were transfected with siRNA targeting *ERBB3* (E3 Sitha Custom Select siRNA; Ambion) or AllStars negative control siRNA (Qiagen) using Lipofectamine 2000 (Invitrogen) following manufacturers’ instructions.

### Western Blotting

For ErbB3 protein expression analysis, RD transfectants were lysed 24 hours after transfections and analyzed by Western blotting with anti-ErbB3 (sc-285; Santa Cruz Biotechnology, Inc.), as previously described [16]. Loading was controlled by reblotting with anti-actin (sc-1616; Santa Cruz Biotechnology, Inc.).

### Immunohistochemistry

Four-micrometer thin paraffin sections representing pediatric alveolar rhabdomyosarcoma, pediatric Ewing’s sarcoma, or normal adult stomach were analyzed for ErbB3 or EphA2 expression using sc-285 (1∶100 dilution) or sc-924 (1∶250 dilution) (Santa Cruz Biotechnology, Inc.) primary antibodies, respectively. After treatment with ChemMate (DakoCytomation Denmark A/S) antigen retrieval solution at pH 9.0, the staining was carried out using automated immunostainer (Lab Vision Autostainer), and Brigth Vision Poly-HRP-anti Ms/Rb/Rt IgG kit (Immuno Logic).

### MTT Proliferation Assay

To address the effect of *ERBB3* on cell growth, RD cells were plated onto 96-well plates (Nunc) at a density of 5000–6000 cells/well in DMEM (Gibco) containing 10% FCS (Biochrom) 24 hours after transfections with plasmid or siRNA constructs, respectively. The number of viable cells was estimated at indicated time points after plating using CellTiter 96 nonradioactive cell proliferation assay (MTT; Promega).

### Statistical Analyses

Gene expression differences associated with a particular pediatric cancer were measured and ranked using gene tissue index (GTI) outlier statistic, as previously described [17]. Expression values of each gene in a single pediatric tumor (test group) were compared with the expression levels in other pediatric normal and cancer samples combined (reference group). This is a common approach used to find cancer biomarkers based on gene expression. For example, gastrointestinal stromal tumor (GIST) patients with high amplification of *KIT* benefit from c-KIT inhibitors like imatinib [18]. Similarly we wanted to identify drug targets showing specific expression in one pediatric tumor.

The Fisher’s exact test (Cox, 1970) was used to test for the fold-change difference of each gene between the test group and the reference group. To correct for multiple testing, we applied Bonferroni and Benjamin Hochberg multiple testing correction methods for assessing the significance of the outlier differences (corrected *P*<0.05).

Statistical analysis of the MTT proliferation assay was carried out with Student’s independent samples t-test. Six to eight parallel samples were analyzed for each time point, and each experiment was repeated at least twice.

## Results

### Targets of Approved Cancer Drugs in Pediatric Sarcomas

Publicly available Affymetrix microarray data from Genesapiens (www.genesapiens.com) database were analyzed to profile the expression of 24 genes in various malignant (n = 1015) and non-neoplastic (n = 154) pediatric tissue samples. Data from Genesapiens were normalized using the array-generation-based gene centering (AGC normalization algorithm). The AGC normalization method effectively removes platform and batch effects that may result from integrating data from multiple sources, as described in detail by Kilpinen et al. [12].

The 24 genes were selected for the analysis as they are known targets for molecularly targeted drugs currently in use for adult malignancies. A focused heatmap analysis of the 24 selected cancer drug targets in pediatric sarcomas and selected control tissues indicated that a few of the targets were indeed selectively overexpressed in pediatric Ewing’s sarcoma and in embryonal and alveolar rhabdomyosarcoma when compared to normal muscle tissue and hematopoietic cells (Fig. 1). *EPHA2* (encoding EphA2, ephrin type A receptor 2), *VEGFC* (vascular endothelial growth factor-C), and *FKBP1A* (FK506 binding protein 1A) were overexpressed in Ewing’s sarcomas. In contrast, *EGFR* (epidermal growth factor receptor), *VEGFR2* (VEGF receptor 2 = KDR), and *PDGFRA* (platelet-derived growth factor receptor α) were overexpressed in embryonal, and *ERBB3* (ErbB3) in alveolar and unclassified rhabdomyosarcomas (Fig. 1). Overexpression of these genes in the specific sarcoma subtypes was also statistically significant when the whole pediatric Genesapiens database was explored for differential expression using Fisher’s exact test (File S1). Moreover, *EPHA2* and *VEGFC* ranked high in Ewing’s sarcoma, *PDGFRA* in embryonal rhabdomyosarcoma, and *ERBB3* in alveolar and unclassified rhabdomyosarcoma, when an outlier expression analysis of the 24 selected cancer drug targets in pediatric sarcomas across the Genesapiens database was carried out using a non-parametric algorithm GTI, a method designed to detect outlier overexpression levels in subsets of samples [17] (File S1).

### Body-wide Expression and Boxplot Analyses *EPHA2*, *VEGFC*, and *ERBB3*

To further address the relative expression of the candidate target genes in the context of all pediatric tissues, body-wide expression analyses were carried out with all 1169 samples representing various malignant and non-neoplastic pediatric tissues. Body-wide expression profiles for *EPHA2* (Fig. 2A), *VEGFC* (Fig. 3A), and *ERBB3* (Fig. 4A) indicated the most selective overexpression in malignant connective tissues, when compared to all other pediatric cancer and healthy normal tissues. Boxplot analyses providing comparisons within the different pediatric cancer samples indicated selective up-regulation of the mean expression of *EPHA2* and *VEGFC* in Ewing’s sarcoma (Fig. 2B and 3B). High *EPHA2* expression levels were also observed in pediatric osteosarcomas (Fig. 2B) in a small number of samples (n = 3). The overexpression of *ERBB3* seemed to be relatively specific for the alveolar subtype of pediatric rhabdomyosarcoma (Fig. 4B). Fisher’s exact test demonstrated that the relative overexpression *EPHA2* and *VEGFC* in Ewing’s sarcoma was 15- and 17-fold, respectively, when compared to all pediatric cancer and normal samples (*P*<0.001 for both comparisons) (File S1). The relative overexpression of *ERBB3* in alveolar and in unclassified (not otherwise specified) rhabdomyosarcoma samples was 5-fold (*P* = 0.04) and 8-fold (*P*<0.001), respectively (File S1).

### Validation of Expression and Activity of ErbB3 in Rhabdomyosarcoma

While tyrosine kinase inhibitors blocking EphA2 or VEGF-C signaling are already being tested as potential targets for sarcomas [19], [20], no information about the biological role of ErbB3 in pediatric rhabdomyosarcoma is currently available. To determine the expression of ErbB3 protein in a rhabdomyosarcoma cell line RD, and to characterize the specificity of the ErbB3 antibody sc-285 used, Western analyses were carried out. The anti-ErbB3 detected a single major band of approximately 180 kD in parental RD cells (Fig. 5A, lane 1). The intensity of the band was significantly increased by plasmid-mediated *ERBB3* overexpression (Fig. 5A, lane 3 *vs.* 2), and suppressed by RNA interference-mediated *ERBB3* down-regulation (Fig. 5A, lane 5 *vs.* 4). Immunohistochemical analysis with the same anti-ErbB3 demonstrated strong staining in the gland epithelium of normal gastric corpus (Fig. 5B), consistent with the previously described ErbB3-specific staining pattern [21] (www.proteinatlas.org). When ten clinical samples representing pediatric alveolar rhabdomyosarcoma were analyzed for ErbB3 expression, strong cytoplasmic expression was detected in one (Fig. 5C), and weak to moderate expression in two (Fig. S1D), whereas seven samples demonstrated no expression (Fig. 5D; data not shown). Six out of six analyzed Ewing’s sarcoma samples were totally negative for ErbB3 immunoreactivity (Fig. S1C), indicating specificity of ErbB3 overexpression in pediatric rhabdomyosarcoma, and consistent with the *in silico* findings (Fig. 4B). As a positive control for the Ewing’s samples, and again consistent with the *in silico* findings (Fig. 2B), all ten analyzed Ewing’s sarcoma samples demonstrated variable degrees of immunohistochemical EphA2 expression (Fig. S1A).

Finally, to address the effects of both gain-of-function and loss-of-function of *ERBB3* for the growth of rhabdomyosarcoma cells, RD cell transfectants overexpressing *ERBB3* or with siRNA-targeted *ERBB3* (Fig. 5A) were analyzed using MTT assays measuring the amount of viable cells. Overexpression of ErbB3 significantly promoted the growth of RD cells (Fig. 5E), whereas ErbB3 down-regulation resulted in significantly reduced RD cell growth (Fig. 5F). Taken together, these findings indicated that, consistent with the data from the *in silico* approach, ErbB3 protein was expressed in a subpopulation of pediatric alveolar rhabdomyosarcoma patients, and that ErbB3 expressed in RD cells was biologically significant in promoting growth.

## Discussion

The survival rates for many pediatric sarcomas have remained poor for the past decades and novel therapeutics have been slow to enter into pediatric practice.

We found that the gene encoding receptor tyrosine kinase EphA2, the ephrin type A receptor 2, is highly expressed in pediatric Ewing’s- and osteosarcomas. The EphA2 receptor and the ephrinA1 ligand have previously been found to be overexpressed in the majority of human osteosarcomas [22]. Our immunohistochemical analysis demonstrated that EphA2 was also expressed at the protein level in ten out of ten samples representing clinical pediatric Ewing’s sarcoma. Recent studies have indicated Eph receptors and their ephrin ligands roles in promoting tumor formation and progression, as well as in stimulating tumor angiogenesis and metastasis [23]–[25]. Thus, the ephrin/Eph receptor system has emerged as an attractive therapeutic target in a number of aggressive adult tumors including breast, lung, colon, kidney, ovarian, and prostate cancer, as well as melanoma and neuroblastoma [26], [27].

Dasatinib is a tyrosine kinase inhibitor that targets several tyrosine kinases, including the Eph receptors (EphA and EphB), PDGFRβ, c-Kit, Src family kinases, and the Bcr-Abl fusion protein [28]. It has been approved for use in Philadelphia chromosome-positive chronic myelogenous leukemia. Consistent with our finding, dasatinib has also been shown to inhibit migration of Ewing’s sarcoma, osteosarcoma and rhabdomyosarcoma cell lines, and selectively block the survival of Ewing’s- and osteosarcoma cells [19]. Moreover, dasatinib has been shown to suppress growth of three out of five osteosarcoma tumor xenografts [29]. Thus, dasatinib could be tested as novel therapeutics for pediatric Ewing’s- and osteosarcomas.

In addition to *EPHA2*, we found that *VEGFC* expression was exceptionally high in pediatric Ewing’s sarcomas. Possibly due to the low local concentrations of the soluble VEGF-C protein *in vivo*, or to the characteristics of the antibodies tested, we were however not able to immunohistochemially address the expression of VEGF-C protein in paraffin-embedded tissue sections (data not shown). VEGF-C is a member of the VEGF family of ligands, that regulates both angiogenesis and lymphangiogenesis by interacting with the VEGF receptors VEGFR-2 and VEGFR-3 [30]. The growth, migration and dissemination of sarcoma cells may also be dependent on angio- and lymphangiogenesis [31]–[33].

Broad-spectrum kinase inhibitors targeting VEGFRs, such as sunitinib, semaxanib (SU5416) and SU6668 and the VEGF-A antibody bevacizumab, have been shown to inhibit growth of Ewing’s sarcoma in mouse xenograft models [34], [35]. Cediranib (AZD2171), a VEGFR family inhibitor that also blocks VEGF-C-induced VEGFR-3 activity [36], inhibits growth of pediatric sarcoma xenografts (Ewing’s-, rhabdoid- and osteosarcomas) in mice [37]. A recent phase I trial of cediranib in children and adolescents also demonstrated responses in patients with Ewing’s- or synovial sarcoma [20]. In addition, clinical trials with broad spectrum inhibitors sorafenib and pazopanib, that both also target VEGFR-3, have shown improved progression-free survival rates in adult angio-, leimomyo- and synovial sarcomas [38], [39]. Of these drugs, sunitinib has been approved for renal cell carcinoma and gastrointestinal stromal tumors, bevacizumab for colorectal cancer, non-small cell lung cancer, glioblastoma, renal cell carcinoma and breast cancer, and pazopanib for renal cell carcinoma. Semaxanib and cediranib are presently in phase I clinical trials. Taken together, broad spectrum VEGFR inhibitors, currently in use or tested for adult cancer patients, may slow progression of pediatric sarcomas.

Finally, we found that *ERBB3* (*HER3*) transcript is highly expressed in pediatric alveolar rhabdomyosarcomas. Down-regulation of ErbB3 levels also led to suppression of rhabdomyosarcoma cell growth *in vitro*. Moreover, ErbB3 protein was present in three out of ten immunohistochemically analyzed clinical samples. It is of note, that both the *in silico* as well as the *in vivo* approaches indicated significant quantitative variation in ErbB3 expression between individual samples. While the percentage of ErbB3-positive alveolar rhabdomyosarcomas needs to be verified in larger clinical series, our findings suggest that ErbB3 targeting could be effective only for a subpopulation of patients, and ErbB3 immunohistochemistry considered as a predictive test. ErbB3 belongs to the human epidermal growth factor receptor family of receptor tyrosine kinases together with EGFR (ErbB1, HER1), ErbB2 (HER2), and ErbB4 (HER4) [5], [40]. Both EGFR and ErbB2 have been established as clinically relevant drug targets, and the therapeutic antibodies trastuzumab, cetuximab and panitumumab, as well as the tyrosine kinase inhibitor erlotinib, gefitinib and lapatinib, are currently used in the therapy of adult patients with breast, gastric, colorectal, head and neck, lung, or pancreatic cancer [5], [41]. As ErbB receptors have a tendency to form heterodimers some of these drugs indirectly also affect ErbB3 signaling [42]. In addition, pharmaceutical companies are currently in the process of developing therapeutic antibodies that would directly block the function of ErbB3.

Two phase I studies with gefitinib, an EGFR inhibitor, suggested that gefitinib is safe and well tolerated in children with solid tumors, but had limited clinical activity. However, in the two studies one out of three patients with Ewing’s sarcoma had a partial response [43], [44]. Erlotinib, another EGFR inhibitor, has also been reported to be well tolerated in children with solid tumors, but to lack significant antitumor activity against rhabdomyo- or osteosarcomas [44], [45]. Currently, no data are available about the activity of ErbB2- or directly ErbB3-targeted therapy in pediatric sarcoma.

In summary, we found that *EPHA2* and *VEGFC* are highly expressed in pediatric Ewing’s sarcomas and *ERBB3* in pediatric rhabdomyosarcomas. Thus, currently available targeted cancer drugs such as inhibitors of the EphA2, VEGF or ErbB receptors could be tested as novel therapeutics for pediatric bone tumors. These cancer types are currently treated with cytotoxic chemotherapy with limited clinical effect and a wide range of adverse effects. In the future, identification of druggable targets in pediatric tumors may enable customizing the most effective treatment for individual patients while minimizing unnecessary side effects. However, characterization of the optimal drug targets and the design of future clinical investigations remain a great challenge.

## Supporting Information

Figure S1 **EphA2 but not ErbB3 is expressed in pediatric Ewing’s sarcoma.** Immunohistochemical analysis demonstrates that EphA2 is expressed in pediatric Ewing’s sarcoma (**A**), as well as in the positive control tissue, adult human stomach (**B**). In contrast, Ewing’s sarcoma is negative for ErbB3 (**C**), while a sample of pediatric alveolar rhabdomyosarcoma demonstrates ErbB3-positivity (an example of weak staining) (**D**).
(PDF)

File S1 **Statistical analysis of drug target expression in pediatric sarcomas.**
(XLS)
