# Supplementary figures and images for: Systemic Analysis of Gene Expression Profiles Identifies ErbB3 as a Potential Drug Target in Pediatric Alveolar Rhabdomyosarcoma
Source: PLoS One. 2012 Dec 5;7(12):e50819. doi: 10.1371/journal.pone.0050819 (PMC3515522; doi:10.1371/journal.pone.0050819)

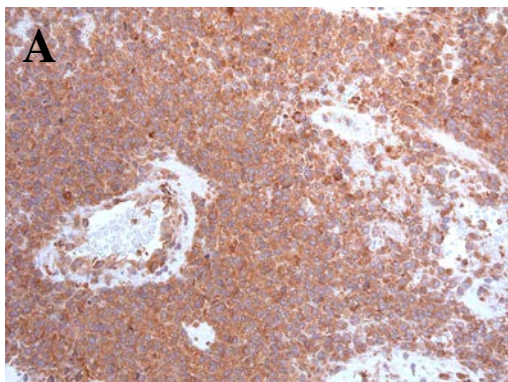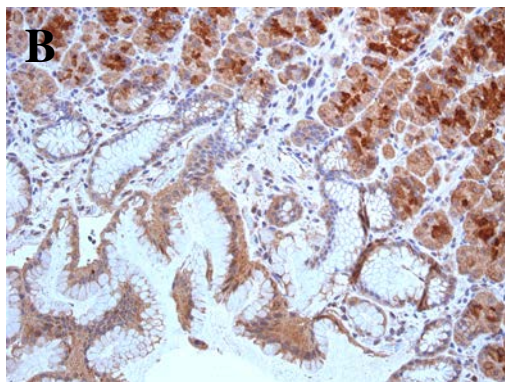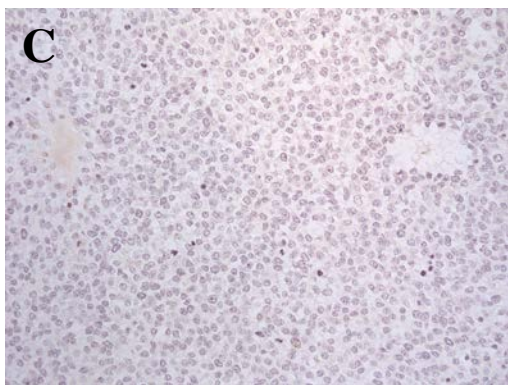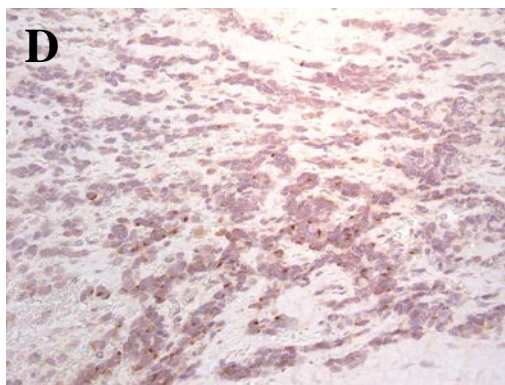

Supplement: Figure S1 — EphA2 but not ErbB3 is expressed in pediatric Ewing’s sarcoma. Immunohistochemical analysis demonstrates that EphA2 is expressed in pediatric Ewing’s sarcoma (A), as well as in the positive control tissue, adult human stomach (B). In contrast, Ewing’s sarcoma is negative for ErbB3 (C), while a sample of pediatric alveolar rhabdomyosarcoma demonstrates ErbB3-positivity (an example of weak staining) (D). (PDF) [file pone.0050819.s001.pdf]
